# Membranes for Modelling Cardiac Tissue Stiffness In Vitro Based on Poly(trimethylene carbonate) and Poly(ethylene glycol) Polymers

**DOI:** 10.3390/membranes10100274

**Published:** 2020-10-03

**Authors:** Iris Allijn, Marcelo Ribeiro, André Poot, Robert Passier, Dimitrios Stamatialis

**Affiliations:** 1 Bioartificial Organs, Biomaterials Science and Technology, University of Twente, 7500 AE Enschede, The Netherlands; d.stamatialis@utwente.nl; 2 Applied Stem Cell Technologies, University of Twente, 7500 AE Enschede, The Netherlands; m.ribeiro@utwente.nl (M.R.); robert.passier@utwente.nl (R.P.); 3 Biomaterials and Regenerative Medicine, Biomaterials Science and Technology, University of Twente, 7500 AE Enschede, The Netherlands; a.a.poot@utwente.nl

**Keywords:** poly(trimethylene carbonate), poly(ethylene glycol), cardiotoxicity, drug screening, cardiomyocyte contraction

## Abstract

Despite the increased expenditure of the pharmaceutical industry on research and development, the number of drugs for cardiovascular diseases that reaches the market is decreasing. A major issue is the limited ability of the current in vitro and experimental animal models to accurately mimic human heart disease, which hampers testing of the efficacy of potential cardiac drugs. Moreover, many non-heart-related drugs have severe adverse cardiac effects, which is a major cause of drugs’ retraction after approval. A main hurdle of current in vitro models is their inability to mimic the stiffness of in vivo cardiac tissue. For instance, poly(styrene) petri dishes, which are often used in these models, have a Young’s modulus in the order of GPa, while the stiffness of healthy human heart tissue is <50 kPa. In pathological conditions, such as scarring and fibrosis, the stiffness of heart tissue is in the >100 kPa range. In this study, we focus on developing new membranes, with a set of properties for mimicry of cardiac tissue stiffness in vitro, based on methacrylate-functionalized macromers and triblock-copolymers of poly(trimethylene carbonate) and poly(ethylene glycol). The new membranes have Young’s moduli in the hydrated state ranging from 18 kPa (healthy tissue) to 2.5 MPa (pathological tissue), and are suitable for cell contraction studies using human pluripotent stem-cell-derived cardiomyocytes. The membranes with higher hydrophilicity have low drug adsorption and low Young’s moduli and could be suitable for drug screening applications.

## 1. Introduction

To date, cardiovascular diseases (CVD) are still the major cause of death worldwide and have the highest estimated health costs (200 billion dollars annually) in the United States. CVD are caused by both external factors (e.g., smoking, diet and inactivity) and by genetic inheritance [1]. Unfortunately, the current drugs against CVD are very limited, as the development of new and better drugs has been hampered by the lack of proper CVD models. Animal models not only have severe limitations in human predictability, but are also expensive and do not allow for high-throughput screening. Progress in human pluripotent stem-cell-based in vitro models have the potential to create a paradigm shift in drug discovery. However, current culture methods using hard plastic or glass do not represent the in vivo environment, which consequently hampers the contractile output of cardiomyocytes (CMs). This is of primary importance for studying cardiac cells, since increased stiffness in cardiac tissue has major implications in CVD.

The Young’s modulus of a healthy human heart is below 50 kPa and increases under pathological conditions, such as scarring and fibrosis, to more than 100 kPa [2,3]. Stiffness of the cell substrate influences the contraction behavior of the CMs; the stiffer the material, the more ‘stress’-like beating is displayed [4].

Current well-plates for testing are convenient for high-throughput screening. However, they are typically made of poly(styrene), which has a Young’s modulus in the GPa range, two orders of magnitude higher than desired. Hydrogels (e.g., poly(acrylamide)) which mimic the soft nature of cardiac tissue very well, tend to be brittle and therefore difficult to upscale to the numbers required for high-throughput screening. Poly(dimethylsiloxane) (PDMS), which is generally used in microfluidic drug screening applications, can be tuned in a wide range of Young’s moduli [5]. However, an important drawback is its high small-molecule-absorbing and -adsorbing nature [6,7,8,9], making often efficacy, toxicity or sequential drug testing unreliable.

This study aims to develop novel, thin and transparent membranes for live cell imaging with a range of properties for mimicry of cardiac tissue stiffness in vitro, based on methacrylate-functionalized macromers [10] and triblock-copolymers of poly(trimethylene carbonate) (PTMC) and poly(ethylene glycol) (PEG). PTMC is tough, flexible and amorphous, and is enzymatically degraded through surface erosion without acidic degradation products and without losing its mechanical properties [11]. PEG is non-toxic, non-immunogenic and highly soluble in water, giving it a comparable Young’s modulus to physiological cardiac tissue. However, due to its hydrophilic nature, PEG is non-cell-adhesive and the soft PEG hydrogels are too brittle to be applied in larger-scale cell-screening systems.

In this work, we hypothesize that the combination of PTMC and PEG could result in highly tunable membranes, with Young’s moduli in the physiological and pathological cardiac tissue range. This will enable drug efficacy studies on CMs in both healthy and diseased conditions.

## 2. Materials and Methods

### 2.1. Chemicals

PEG (*M*n = 10 kg/mol), tin(II) 2-ethylhexanoate (Sn(Oct)_2_), hydroquinone (HQ), triethylamine (TEA), methacrylic anhydride (MAAh), deuterated chloroform, 1,6-hexanediol, 2-hydroxy-4′-(2-hydroxyethoxy)-2-methylpropiophenone (Irgacure 2959), tris(hydroxymethyl)aminomethane (TRIS), Matrigel, verapamil hydrochloride (CAS RN: 52-53-9) and 3,4-dihydroxy-L-phenylalanine (L-DOPA) were purchased from Sigma-Aldrich (Zwijndrecht, The Netherlands). Propylene carbonate (PC), ethanol absolute and calcium hydride were obtained from Merck Millipore (Darmstadt, Germany). Methanol, dichloromethane (DCM) and diethyl ether were purchased from VWR (Amsterdam, The Netherlands). Trimethylene carbonate (TMC) was obtained from Huizhou ForYou Medical Devices Co (Huizou, China). Essential 8 medium was purchased from Thermo Fisher (Naarden, The Netherlands), Wnt activator CHIR99021 (1.5 µmol/L) from Axon Medchem (Groningen, The Netherlands), Activin-A (20 ng/mL) from Miltenyi Biotec (Bergisch Gladbach, Germany) and BMP4 (20 ng/mL) and XAV939 (5 µmol/L) from R&D Systems (Abingdon, UK).

### 2.2. Synthesis and Characterization of Dimethacrylate-Functionalized PTMC, PEG and PTMC-PEG-PTMC Macromers

**Figure 1:**
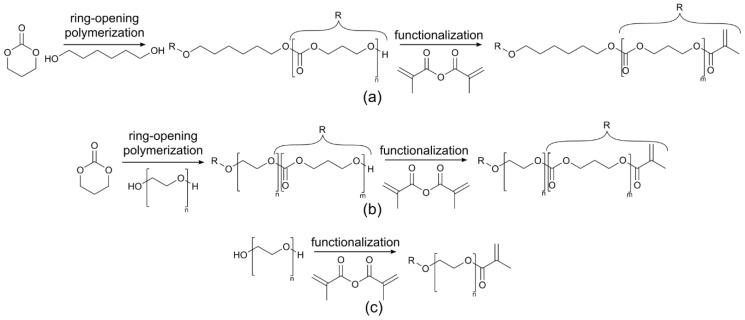
Ring-opening polymerization and functionalization schematic for photo-crosslinkable macromers. (**a**) Hexanediol was used as an initiator for the ring-opening polymerization of trimethylene carbonate (TMC), resulting in two-armed poly-TMC (PTMC), which was functionalized with MAAh, giving PTMC-dMA. (**b**) Commercial polyethylene glycol (PEG) (*M*n = 10 kg/mol) was used as an initiator for TMC polymerization, resulting in a triblock-copolymer, which subsequently was functionalized into PTMC-PEG-PTMC-dMA. (**c**) Commercial PEG (*M*n = 10 kg/mol) was functionalized, resulting in PEG-dMA.

Linear macromers were synthesized by ring-opening polymerization of TMC (Figure 1) as described previously [12,13] with slight modifications. Briefly, polymerization was conducted in an argon atmosphere at 130 °C for three days with Sn(Oct)_2_ as a catalyst. Hexanediol (0.1 mmol/g monomer to obtain an oligomer with *M*n = 10 kg/mol) was used as an initiator for PTMC, whereas PEG with *M*n = 10 kg/mol (0.95 g/g monomer) was used as an initiator to obtain PTMC-PEG-PTMC triblock-copolymer. Cooled-down oligomers and commercial PEG (*M*n = 10 kg/mol) were functionalized in DCM (dried over calcium hydride and distilled) by reaction with MAAh (6 mol/mol initiator) in the presence of TEA (6 mol/mol initiator) and HQ (0.1% w/w to TMC) for five days at room temperature (RT). Subsequently, the functionalized macromers were purified by precipitation in ice cold methanol (for PTMC-dimethacrylate (dMA)) or in ice cold diethyl ether (for PEG-dMA and PTMC-PEG-PTMC-dMA). The obtained macromers were dried in the dark at RT overnight and in vacuo for one week until constant weight was achieved. The (non-)functionalized macromers were dissolved in deuterated CHCl_3_ to determine the TMC conversion, the number average molecular mass (*M*n) and the degree of functionalization (df) by ^1^H-NMR (Bruker Ascend 400/Avance III 400 MHz NMR spectrometer). The TMC conversion from the ^1^H-NMR spectrum was calculated by comparing the area of the TMC peak at 4.46 ppm with the area of the PTMC peak at 4.24 ppm. The *M*n for PTMC in PTMC-dMA and for PTMC in PTMC-PEG-PTMC-dMA was determined by comparing the area of the PTMC methylene peak at 4.24 ppm with the area of the initiator peak (hexanediol at 1.42 ppm and PEG at 3.61 ppm, respectively). Methacrylation of PTMC, PEG and PTMC-PEG-PTMC was confirmed by the double bond proton peaks at 5.58 and 6.11 ppm, and the df was determined by comparing the area of these peaks with the initiator peak at 1.42 ppm.

### 2.3. Polymeric Network Formation—Membrane Fabrication

Photo-crosslinked networks of PTMC-dMA, PEG-dMA, PTMC-PEG-PTMC-dMA and mixtures thereof were prepared. To this end, a 25% w/w polymer (PTMC-dMA and PEG-dMA) or 40% w/w (PTMC-PEG-PTMC-dMA) solution in PC was prepared, containing 4% w/w (to the total polymer amount) photo initiator Irgacure 2959. The polymer solutions were stirred at 50 °C to get a clear viscous solution and were mixed in various ratios. A square frame of parafilm was stuck onto a borosilicate glass plate. Subsequently, the polymer solution was poured on the glass in the middle of the parafilm frame and was sandwiched with a second borosilicate glass plate to form a polymer solution layer in between. The films were photo-crosslinked in solution under N_2_ for 30 min at 280 nm and 13.5 mW/cm^2^ (Ultra-Lum Electronic Ultraviolet Crosslinker with a g15 UV-C lamp, USHIO America Inc., Cypress, CA, USA). The photo-crosslinked membranes were swollen in PC, slowly extracted with increasing ethanol concentrations and left to dry at RT.

### 2.4. Characterization of Photo-Crosslinked Networks

The gel content of the photo-crosslinked networks was determined as a measure of successful network polymerization. Since the films were photo-crosslinked in solution and PC (boiling point = 242 °C) does not evaporate, the initial weight of the film before extraction was corrected for PC (m_corr_) (Equation (1)). The buffer uptake of the dry photo-crosslinked networks was determined by weighing the dry and extracted networks (m_d_) and the wet networks (m_w_) after swelling 24 h in PBS (pH 7.4) at RT and blotting dry (Equation (2)).

gel content = (m_d_)/(m_corr_) × 100% (1)

buffer uptake = ((m_w_ − m_d_)/(m_d_)) × 100% (2)

The mechanical properties of the photo-crosslinked networks were assessed when fully swollen in PBS, as this is the state of the membranes when the CMs will attach. For this, at least five replicates (100 × 5 mm^2^) per membrane were measured in a tensile tester (Zwick Z020, Zwick/Roell, Ulm, Germany) with a 500 N load cell, an initial grip-to-grip separation of 30 mm, and a test speed of 50 mm/min. The Young’s modulus (E in kPa) at 1–4% elongation, maximal stress (σ_max_ in kPa) and elongation at break (ε_max_ in %) were determined. The toughness (W_t_ in N/mm^2^) of the samples was defined as the area under the stress–strain curve.

### 2.5. Verapamil Adsorption to the Membranes and PDMS

For static adsorption experiments, a 4 mM verapamil stock solution in DMSO was diluted with PBS to a working solution of 1 mM. Meanwhile, the membranes, fully swollen in PBS, were cut in circles with a diameter of 1 cm. PDMS (Sylgard 184, ratio 1:10, crosslinker:silicone) was poured as a thin layer of comparable thickness as the membranes in a petri dish and cured at 60 °C for 18 h and was used as a positive control. All samples were put in glass vials containing 2 mL of working solution and put on gentle shaking at 37 °C for 3 h. DMSO was used as a vehicle control and the working solution without any material was used as a control for drug degradation and adsorption to the glass. The membranes were removed after 3 h and the verapamil concentration in the working solution was measured at 280 nm using a spectrophotometer (Tecan Infinite 200 Pro, i-control software). The amount of verapamil adsorbed to 1 mm^2^ membrane or PDMS was calculated and adsorption values were compared.

### 2.6. Cell Culture Plate Assembly and L-DOPA/Matrigel Double Coating

Circular membrane pieces with a diameter of 17 mm were cut from the fully in PBS (pH 7.4) swollen membranes, placed on a glass coverslip (diameter of 22 mm), and attached to the bottom of bottomless 12-well plates (gap diameter of 14 mm, MatTek, Ashland, MA, USA) using high-vacuum grease (Dow Corning). The edges of the coverslips were then sealed watertight using glue (Griffon combi 2-component). Subsequently, the films were UV-sterilized in a laminar flow hood for 30 min. L-DOPA coating was applied to the membranes as described previously [14], with some modifications. Briefly, samples were incubated with 10 mM TRIS buffer at pH 8.5 for at least 1 h. Meanwhile the L-DOPA (2 mg/mL) was dissolved in 10 mM TRIS buffer at pH 8.5 at 37 °C, until the solution turned gray, and filter sterilized. Afterwards, the membranes were incubated with this solution for 20 h in a 37 °C incubator to allow the L-DOPA to polymerize. Unattached L-DOPA solution was washed away three times using Hank’s Balanced Salt Solution (HBSS) and Matrigel (1:200 in DMEM 33133) coating was applied for 2 h at RT and aspirated before adding medium.

### 2.7. Cardiomyocyte Culture on Polymeric Membranes

We have recently generated a double fluorescent reporter mRubyII-ACTN2 and GFP-NKX2.5 (DRRAGN) human embryonic stem cell line, which allows us to visualize beating CMs by live imaging [4]. Human pluripotent stem-cell-derived CMs from this reporter cell line were cultured in BPEL medium until day 20 and were maintained for four more days in our previously published CM medium [15] with 10% horse serum (Gibco). Cells were dissociated using TriplE 1x and plated on the membranes at a density of 70,000 cells/1.5 cm^2^ in CM medium with 10% horse serum. The cells were cultured for five days, with medium refreshment every two days, on the membranes and glass (positive control) in duplicates. Recordings of single cell contractions were done under external pacing at 1 Hz, using a frame rate of 50 frames per second and a 30× magnification in a Nikon microscope.

### 2.8. Contractility Analysis of the Cardiomyocytes

The degree of contraction of CMs was determined from the image sequences using Image J (NIH) for at least ten cells per condition. The surface area of the cells was determined manually in both the contracted and the relaxed state. Subsequently, the difference in surface area was taken as a measure of contraction.

### 2.9. Statistical Analysis

Statistical analysis was performed using a one-way ANOVA test with a Tukey’s Multiple Comparison Test (GraphPad Prism, Version 5.00). All data are shown as mean ± SEM, with n = 5 for the mechanical testing, n = 3 for the adsorption testing and n = 10 for the contractility analysis.

## 3. Results and Discussion

### 3.1. Macromer Synthesis and Properties

Two-armed PTMC and PTMC-PEG-PTMC macromers were obtained by ring-opening polymerization using hexanediol and PEG (*M*n = 10 kg/mol) as initiator, respectively (Figure 1). These products and commercial PEG (*M*n = 10 kg/mol) were subsequently reacted with MAAh to yield photo-crosslinkable macromers (Table 1). The conversion was >97% for both oligomers and all macromers had a df >90%.

### 3.2. The Characteristics of the Photo-Crosslinked Membranes Can Be Tailored by Mixing the Macromers

Flat membranes were prepared: three single macromeric (M1–M3) and three mixed macromeric (M4–M6) (Table 2). For the triblock-copolymer PTMC-PEG-PTMC-dMA, which has a double molar mass compared to PTMC-dMA and PEG-dMA, a concentration of 25% (w/w) did not give a sufficient number of MA-groups to form a macromeric network. Instead, a solution of 40% (w/w) was prepared, which still had a lower number of MA-groups than PTMC-dMA and PEG-dMA, but enough to form a good macromeric network.

To decrease the Young’s modulus of the membranes made from PTMC-dMA (M1) and PTMC-PEG-PTMC-dMA (M3) and to increase the toughness of the membranes made from PEG-dMA (M2), mixed macromeric membranes were prepared. PTMC-dMA was mixed with PEG-dMA in two different weight ratios, 50:50 (M4) and 10:90 (M5), and PTMC-PEG-PTMC-dMA was mixed with PEG-dMA (M6). M4 served as a control for M3, having the same molar ratio of PTMC:PEG in the macromeric networks (Table 2). Furthermore, M6 had a molar ratio of PTMC:PEG close to that of M5. In this way, the effect of the triblock copolymer in the macromeric network on the Young’s modulus could be studied. The gel content of all prepared networks was above 80%, indicating good network formation. M3 had the lowest gel content, which is in line with the lower number of available reactive MA groups to form a network.

The buffer uptake of the dry membranes (Table 3) was dependent on the PEG content. This is not surprising, since PEG is a hydrophilic polymer and hence forms a hydrogel in an aqueous environment. Of the dry membranes, only PTMC was transparent. However, upon swelling in buffer, all membranes were transparent, a desired requirement for live cell imaging.

**Figure 2:**
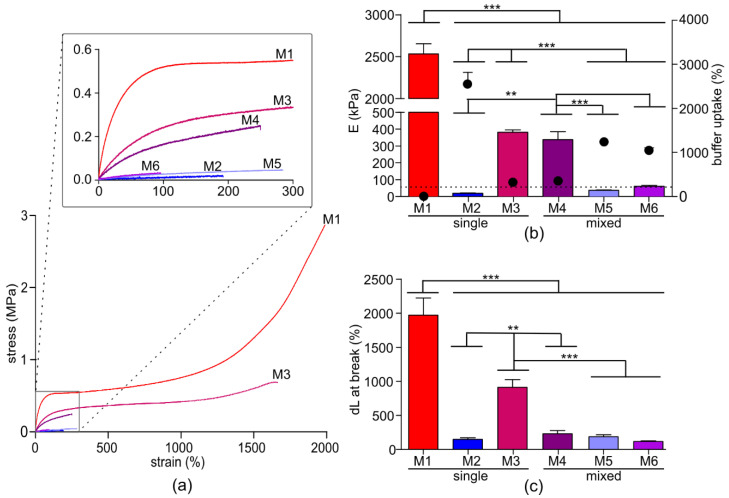
Mechanical properties and buffer uptake of the membranes. (**a**) Stress-strain curves of the membranes, with an inset of the first 300% strain. (**b**) Ashby plot of the Young’s modulus (bars, left Y-axis) vs. the buffer uptake (dots, right Y-axis) of the membranes. The dotted line indicates the maximum physiological stiffness of 50 kPa (**c**) Elongation at break of the membranes. * *p* < 0.05, ** *p* < 0.01, *** *p* < 0.001.

The mechanical properties of the membranes (determined in the swollen state from the stress-strain curves) were inversely proportional to the buffer uptake of the membranes (Figure 2). The Young’s modulus of the membranes is the most important parameter affecting CM contraction. If this exceeds 100 kPa, fibrotic heart tissue is mimicked instead of healthy heart tissue [2]. Another important parameter for the application of membranes using cultured CMs for high-throughput analysis is the toughness. The toughness, determined as the area under the stress–strain curve, is a measure of the energy the macromeric network can absorb before it breaks. In other words, the higher the toughness, the less brittle it is, which is essential for handling membranes for large high-throughput screening systems. Therefore, the ideal membrane as a CM substrate is soft enough to mimic the healthy heart, yet tough enough to integrate in upscaled screening systems.

The Young’s modulus of the six membranes developed here mimicked healthy (M2, M5 and M6) and fibrotic pathological (M1, M3 and M4) heart tissues (Table 3). M1 has a Young’s modulus of 2531 kPa which is consistent with what has been reported before [10,12], has an elongation at break of almost 2000% and is extremely tough. As expected, this membrane is not suitable for mimicking physiological CM contraction behavior. On the other hand, M2 has a suitable stiffness (Young’s modulus is 18 kPa). However, with an elongation at break of only about 145%, it is very brittle and therefore difficult to handle and to use for upscaling. M3 has a Young’s modulus of 381 kPa and an elongation at break of 910%, resulting in a tough hydrogel which is easy to handle. Interestingly, M4 has with 338 kPa a similar Young’s modulus (not significantly different), however, the elongation at break is with 227% significantly lower (*p* < 0.01, **). Therefore, M3 from the triblock-copolymer is more suitable for upscaled high-throughput screening systems. These results suggest that a homogenous distribution of PEG in the macromeric network, which is the case for M3, is beneficial for the toughness without influencing the Young’s modulus. For M4, where two macromers are mixed, the well-defined PTMC/PEG distribution is disturbed by phase separation and the enhanced toughness is lost. Although the Young’s moduli of M5 and M6 are in the physiological stiffness range of cardiac tissue, their toughness is higher than that of M2 (Figure 2b), and therefore M5 and M6 would be more suitable for in vitro screening models.

### 3.3. Verapamil Adsorption to the Membranes

**Figure 3:**
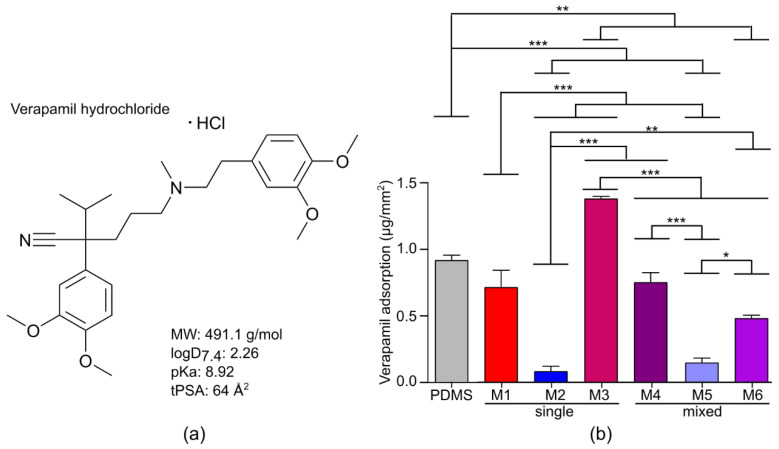
Verapamil adsorption to the membranes. (**a**) Chemical structure of verapamil hydrochloride and important physicochemical properties for drug development and adsorption studies. (**b**) Verapamil adsorption to the uncoated membranes and PDMS as control after 3 h. Data are the results of three experiments and depicted as mean ± SEM.

Drug adsorption to membranes severely restricts drug screening because the dose-response cannot be determined correctly, and hence the efficacy or toxicity of the drug remains largely unknown. It is therefore important that the membranes display no or very low drug adsorption. Here, we evaluated the drug adsorption of a small drug molecule, verapamil, to the six membranes developed, compared to PDMS. Verapamil (Figure 3a), is an Food and Drug Administration (FDA)-approved calcium channel blocker used for the treatment of, among others, high blood pressure, heart arrhythmias and angina [16]. It complies to Lipinski’s Rule of 5 [17], which is a rule of thumb for druggability, and is also small enough to cross the blood–brain barrier [18]. Furthermore, the pKa is above physiological pH, meaning that, in the body (pH 7.4), this drug is positively charged.

The adsorption of verapamil to the swollen membranes was compared to PDMS membranes and was calculated as the total amount (in grams) adhered to the surface area (in mm^2^). PDMS membranes are often used for drug screening applications and high adsorption of small drug molecules by PDMS has been reported, including paclitaxel, ciprofloxacin [6], nifedipine, bepridil and verapamil [9]. Therefore, PDMS was used as a positive control. Very little verapamil adsorbed to the glass vials, nevertheless, the adsorption by the membranes and PDMS was corrected for this. Furthermore, the DMSO, present in the PBS to dissolve the drug, did not exceed the PBS background values.

In general, the adsorption of verapamil to the more hydrophilic membranes (i.e., M2 and M5) was low after 3 h (Figure 3b). Furthermore, it is interesting to note that the most hydrophobic membrane, PTMC (M1), does not have the highest verapamil adsorption per surface area, despite the hydrophobic nature of verapamil (logD_7.4_ of 2.26). The rather high drug adsorption to the PEG-containing membranes is probably due to their high swelling in aqueous conditions, which allows diffusion of the drug within the hydrogel network. Nevertheless, M2, M5 and M6 had significantly lower adsorption than PDMS after 3 h. The adsorption of verapamil to PDMS was similar to that reported elsewhere [6,7,8,9].

In conclusion, the membranes which could mimic the physiological stiffness of cardiac tissue (i.e., M2, M5 and M6) showed low verapamil adsorption, much lower than the PDMS control. M5, in turn, has significantly lower adsorption than M6 and is therefore a good candidate for an upscaled screening platform for drug testing on CMs.

### 3.4. Cardiomyocyte Contraction Behavior Is Influenced by the Stiffness of the Membranes

Six membranes covering both healthy and fibrotic Young’s moduli were used as cell substrates to study CM contraction movement. The main function of CMs is to contract synchronously in order to generate pressure inside the heart cavities and consequently pump the blood through the circulatory system. In order to mimic this function in vitro, the substrate of the CMs needs to be compliant enough to allow the CMs to fully contract. This compliance is related to the Young’s modulus of the substrate. Glass, which has a Young’s modulus of more than 50 GPa, was used as a negative control. Since the membranes had no pattern or other cues, the CMs would take their shape triggered only by matrix elasticity. The thickness of all membranes was between 180 and 300 µm, and was selected as such for the CMs not to feel the underlying glass coverslips [19].

**Figure 4:**
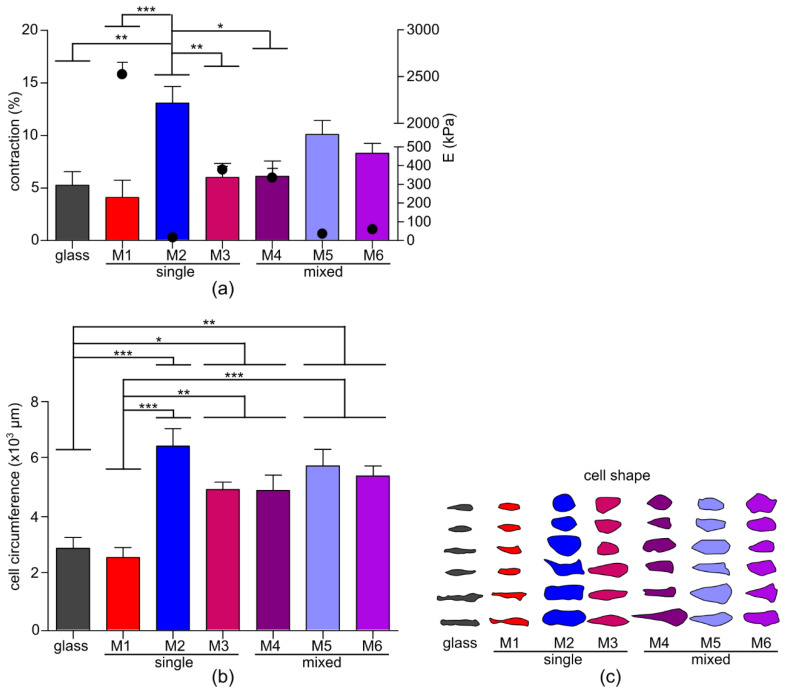
Cardiomyocyte (CM) contraction behavior and shape. (**a**) Degree of contraction of the CMs (bars, left Y-axis) in relation to the Young’s modulus (dots, right Y-axis) shows an increased contraction for a decreased Young’s modulus. (**b**) Cell-spreading circumference of the relaxed CMs is significantly lower for the stiff substrates glass and PTMC (M1). (**c**) Shape as defined by the CM circumferences. * *p* < 0.05, ** *p* < 0.01, *** *p* < 0.001 (Supplementary Video).

The culture medium of the CMs does not contain extracellular matrix (ECM) proteins, therefore a Matrigel coating was applied first to the membranes in order for the CMs to adhere. As expected, the hydrophilic and inert nature of the PEG [20] in the networks made the surface non-adherent for the Matrigel layer. Consequently, CMs only attached to the glass and PTMC membranes. There are multiple strategies to make PEG hydrogels cell adherent using bioactive molecules such as the arginylglycylaspartic acid(RGD)-peptide or making them sensitive to enzyme activity [20]. However, these strategies require modifications during synthesis and therefore are less suitable for block-copolymers, mixed macromer networks or of-the-shelf use. Here, to obtain Matrigel adhesion to the membranes, we applied L-DOPA, derived from an adhesive protein from the common blue mussel, *M. edulis* [21] to polymerize on the membrane surface and act as a glue for Matrigel anchoring, as described previously [14]. In this manner, the Matrigel coating was successfully applied and CMs adhered to all six membranes and to the glass. Furthermore, we observed no differences in the number of beating CMs across the different substrates. Live cell imaging was performed on single beating CMs and their contraction movement was recorded. The surface circumference of the CMs in both the relaxed and the contracted state was measured to obtain information on the contraction motion of the CMs, as well as on the cell size and shape (Figure 4).

Our results show that the contraction motion of the CMs increased with decreasing substrate stiffness. A significant difference in contraction could be observed for M2 vs. glass, M1, M3 and M4, indicating that a low Young’s modulus resulted in high CM contraction motion (Figure 4a). This means that high substrate stiffness generates too much resistance against the contraction motion of the CMs, while at physiological substrate stiffnesses, the CMs increase their contraction motion. In relation to the in vivo situation, the restraint of CM contraction by increased cardiac wall stiffness can lead to decreased stroke volume and, consequently, a decrease in cardiac output. It has been described that CMs counter this restraint by building more contractile sarcomere myofilaments to overcome the opposing resistance enforced by a high cardiac wall stiffness, further increasing the wall stiffness in a self-propagating cycle [22]. Such a cycle has a pivotal role in the development and the progression of cardiac diseases such as hypertrophic and dilated cardiomyopathies and heart failure [23,24,25].

The adhesion area of the CMs was determined in the relaxed state and showed a significantly lower area for both the glass and PTMC substrates (Figure 4b). A clear trend was visible for increasing cell circumference with decreasing Young’s modulus. The shape of the CMs changed from thin elongated for the stiff substrates (i.e., glass and M1) to a rounder and pleiomorphic shape for the softer substrates (i.e., M2, M5 and M6), keeping the major axis length approximately the same, but with increasing width (Figure 4c). The two stiffest substrates (i.e., glass and M1) have, as a consequence of this, an increased aspect ratio. In an earlier study, a similar trend in aspect ratio increase towards the more pathological culture conditions was observed, but with a lower starting aspect ratio [26]. Further direct comparison of these results with other studies is difficult to make, because often the CMs are grown on patterned surfaces with a preset aspect ratio [26,27], or without a specified Young’s modulus of the material [26,28,29,30,31]. Nevertheless, our results are in accordance with studies which report that CMs have a rounder and pleiomorphic shape when cultured on a softer surface [2,32] in comparison to a more elongated CM when cultured on stiffer materials [2], when no preset pattern is applied [29,30].

## 4. Conclusions

Lack of human predictive models is one of the main causes for the very low number of CVD drugs being developed. Novel in vitro drug-screening platforms mimicking cardiac tissue properties are essential for drug discovery in CVD. In this study, we prepared six thin and transparent membranes based on methacrylate-functionalized macromers of PTMC, PEG and the triblock-copolymer PTMC-PEG-PTMC. M1 (PTMC) was very tough, but too stiff for cardiac applications, whereas M2 (PEG) was in the physiological range of cardiac tissue stiffness, but quite brittle for upscaled in vitro drug-screening platforms. Interestingly, although only applicable for mimicking pathological cardiac conditions, M3 (PPP) and M4 (PTMC50:PEG50) had a similar PEG/PTMC ratio with a similar Young’s modulus and comparable CM contraction behavior. While the toughness of M3 was significantly higher, and therefore may be more suitable for upscaling purposes than M4, the verapamil adsorption to M3 was high as well, making it less suitable for drug screening. M5 (PTMC10:PEG90) had a stiffness close to that of physiological cardiac tissue and was still tough enough for handling and upscaling. Furthermore, this membrane had very low verapamil adsorption and the CMs showed healthy contraction behavior. Therefore, it is a good candidate for the in vitro mimicry of cardiac tissue for drug screening applications.

## Figures and Tables

**Table 1:** Macromer properties. TMC conversion and number average molar masses (*M*n) for PTMC-containing macromers and degree of functionalization (df) of all macromers as determined by ^1^H-NMR. The *M*n for PEG as was provided by the manufacturer.

Macromer	TMC Conversion (%)	df (%)	*M*n (kg/mol)		Molar Ratio PTMC:PEG
			PTMC	PEG	
PTMC-dMA	99.1	91.6	9.5	-	-
PEG-dMA	-	93.5	-	10.0	-
PTMC-PEG-PTMC-dMA	97.4	94.8	10.1	10.0	50:50

**Table 2:** Membrane composition. The gel content is the mean ± SEM for at least three networks.

Membrane Identifiers		Network Composition		Molar Ratio		Reactive Groups	Gel Content
#	Name	macromer 1 (mm1)	macromer 2 (mm2)	mm1:mm2	PTMC:PEG	mol MA/g sol	(%)
M1	PTMC	PTMC-dMA	-	-	-	4.8 × 10^−5^	94 ± 1
M2	PEG	PEG-dMA	-	-	-	4.6 × 10^−5^	96 ± 2
M3	PPP	PTMC-PEG-PTMC-dMA	-	-	50:50	3.7 × 10^−5^	84 ± 1
M4	PTMC50:PEG50	PTMC-dMA	PEG-dMA	50:50	50:50	4.8 × 10^−5^	90 ± 4
M5	PTMC10:PEG90	PTMC-dMA	PEG-dMA	10:90	10:90	4.7 × 10^−5^	93 ± 1
M6	PPP26:PEG74	PTMC-PEG-PTMC-dMA	PEG-dMA	26:74	13:87	4.4 × 10^−5^	88 ± 2

**Table 3:** Summary of the membrane characteristics. Reported values are mean ± SEM for at least five measurements.

Membrane		Buffer Uptake	Young’s Modulus	dL at Break	Max Stress	Toughness
#	Name	(%)	(kPa)	(%)	(kPa)	(N/mm^2^)
M1	PTMC	0.8 ± 0.3	2531 ± 120	1969 ± 253	2312 ± 335	2194 ± 423
M2	PEG	2553 ± 257	18 ± 2	145 ± 23	14 ± 1	1.2 ± 0.3
M3	PPP	319 ± 8	381 ± 14	911 ± 113	431 ± 50	370 ± 55
M4	PTMC50:PEG50	351 ± 9	338 ± 47	227 ± 50	286 ± 46	27 ± 6
M5	PTMC10:PEG90	1240 ± 21	37 ± 3	222 ± 44	33 ± 3	4 ± 1
M6	PPP26:PEG74	1053 ± 53	61 ± 6	109 ± 11	54 ± 7	4 ± 1

## Supplementary Materials

The following are available online at https://www.mdpi.com/2077-0375/10/10/274/s1, Video S1: Cardiomyocyte contraction behavior recordings. From top to bottom: glass, single (M1–M3) and mixed (M4–M6) macromeric membranes. The recordings are representative for all measurements and the scale bar is applicable to all CMs frames.
